# The use of color-changeable chewing gum in evaluating food masticability

**DOI:** 10.1007/s41999-023-00916-5

**Published:** 2024-01-12

**Authors:** Toshihiro Yashiro, Shinichi Wada, Nobuyuki Kawate

**Affiliations:** 1 Department of Rehabilitation Medicine, Moriyama Rehabilitation Clinic, 1-11-17 Nishi-Nakanobu, Shinagawa-ku, Tokyo, 142-0054 Japan; 2 Department of Rehabilitation Medicine, Showa University School of Medicine, 2-1-1 Fujigaoka, Aoba-ku, Yokohama-shi, Kanagawa 227-8518 Japan

**Keywords:** Color-changeable chewing gum, Color scale, Geriatrics, Mastication, Nutrition

## Abstract

**Purpose:**

We assess masticatory performance using color-changeable chewing gum, which shifts from yellow-green to red upon chewing. Previous studies used a colorimeter to reveal the cut-off a* values of the gum’s color (a* represents the degree of color between red and green) to determine which foods are masticable (masticable meat, masticable soft side dish, and inadequate masticatory performance). Since a more convenient method is required, we examined the validity and reliability of visual measurement using a color scale to indicate masticable foods by observing the chewing gum’s color.

**Methods:**

Fifty patients (male, 48%), aged ≥ 65 (mean, 82.6) years were enrolled. Using color-changeable chewing gum, three groups were formed according to the cut-off a* values: masticable meat, masticable soft side dish, and inadequate masticatory performance. For the color scale classification, one physiatrist and one dietitian simultaneously, individually, and visually determined the chewing gum color and classified it into one of the three groups. Criterion-related validity was evaluated using the kappa coefficient of agreement between the results from the two methods. Inter-rater reliability was examined using the kappa coefficient of agreement between the three groups judged by the two raters on a color scale.

**Results:**

The kappa coefficients of the agreement for the classifications were 0.908 and 0.909 for the physiatrist and dietitian, respectively. The inter-rater agreement between two raters on the color scale was 0.938 for the kappa coefficient.

**Conclusion:**

The color scale indicated masticable foods without a colorimeter. This method can be useful in older adult facilities and homes.

## Key summary points

**Aim:**

Our study aimed to examine the validity and reliability of visual measurement by assessing chewing gum color, using an existing color scale compared to that assessed using a colorimeter to determine masticable foods.

**Findings:**

The assessment of masticatory performance by the color scale was in agreement with that by colorimeter. The inter-rater reliability was also high.

**Message:**

Visual inspection using a color scale makes it possible to determine masticable food without the use of a colorimeter.

## Introduction

Approximately 15% of all older adults who are independent in their daily activities at home reportedly have dysphagia [1]. Among nursing home residents who were screened and evaluated by a speech-language-hearing therapist for food intake problems, approximately 50% had dysphagia; moreover, nearly 80% of these individuals were not tested despite having dysphagia [2].

The process of chewing and swallowing food is very complex. The skill required to break down solid food, mix it with saliva, collect it into a cohesive bolus, and transport it to the posterior of the oral cavity for swallowing can be challenging to nearly impossible for individuals with oropharyngeal dysphagia. Since general clinicians and caregivers usually do not evaluate all these skills, they tend to prefer to provide safe meal forms with an insufficient evaluation. As a result, unnecessary mechanically altered or pureed food might be provided to older patients, which might have a negative impact on their quality of life [3].

To the best of our knowledge, no method has been introduced to determine masticable foods without the use of expensive instruments. The Test of Masticating and Swallowing Solids has been introduced as a masticatory performance assessment method to measure chewing time, number of masticatory cycles, and number of swallows while the participant ingests cracker [4]. Although other simple methods for evaluating masticatory performance are known, they can evaluate the degree of masticatory performance but cannot determine the meal forms that can be masticated [4–6]. Therefore, there is a need to develop a screening test that is less burdensome and can be performed at the bedside to accurately identify masticatory performance in older persons requiring long-term care.

Masticatory performance can be confirmed with color-changeable chewing gum that changes yellow-green to red when chewed, which is indicated by a colorimeter using the a* value (a* represents the degree of color between red and green) [7]. A previous study has shown a cut-off a* value that determines whether meals (boiled rice, ginger-fried pork loin, boiled fish-paste, rice cracker) are masticable [8]. In that study, a food bolus texture at the swallowing threshold could be regarded as suitable for swallowing if it satisfied three criteria: (1) under 15,000 N/m^2^ in hardness, (2) under 1000 J/m^2^ in adhesiveness, and (3) between 0.2 and 0.9 in cohesiveness. The food bolus texture that is suitable for swallowing was defined as masticable. Therefore, using color-changeable chewing gum, a* values measured by a colorimeter could be divided into three groups according to the following cut-off a* values: masticable meat: > 28.7, masticable soft side dish: 21.2–28.6, and inadequate masticatory performance: < 21.1 [8]. This approach may lead to reductions in unnecessary mechanically altered or pureed food for an older adult who can eat pureed food and safely be provided palatable food. Although this method was able to determine masticable meal forms, measuring the a* value requires a colorimeter to assess the gum color after chewing. The use of a colorimeter is simple and requires no prior training; however, the device is expensive (334,800 Japanese yen or 2443 euros, conversion rate: 1 euro = 137.03 yen when launched on October 24, 2014) and not widely available.

A color scale has been created without using a color-measuring instrument to determine the degree of masticatory performance by visually evaluating the color of the gum simply [7]. The cost of a color scale is 4000 Japanese yen or 29.188 euros as of October 24, 2014, which is only approximately 1/80 of the cost of a colorimeter. It has been shown that there is a correlation between a* values and the color scale; therefore, it can be inferred that a better color scale value indicates better masticatory performance [9]. However, the exact color scale values that indicate meat and soft side dish masticatory performance, as well as inadequate masticatory performance, are currently unknown.

Our study aimed to use a color scale to determine masticable meal form for an older person merely by observing the color of the chewing gum. Widespread use of this method may lead to the provision of appropriate food forms to older persons who are provided inappropriate meal forms.

## Methods

### Setting

The Moriyama Rehabilitation Clinic, located in a residential area in a special ward in Tokyo Metropolis, has 19 beds for inpatients and provides rehabilitation medicine and general medicine for inpatients and outpatients to the local community.

### Participants

All inpatients and daycare attendants of our clinic were assessed for eligibility. The inclusion criterion was ≥ 65 years of age. The exclusion criteria were: (1) inability to obtain the required number of calories by oral intake; (2) inability to follow the instructions required for this study, such as spitting out the gum; (3) poor general condition due to acute illnesses, such as pneumonia; (4) unwilling to provide consent; and (5) deemed inappropriate by the principal investigator or research collaborator. Participants were evaluated as part of a routine assessment and provided similar instructions.

Participant characteristics were confirmed by age, sex, medical history (hypertension, dyslipidemia, diabetes, stroke, Parkinson's disease/Parkinson's syndrome, heart failure), Eichner index (recorded in supporting zones with removable prostheses), repetitive saliva swallowing test, Functional Oral Intake Scale, Seirei dysphagia screening questionnaire (answer of A was defined as suspected dysphagia), and Barthel Index.

This study was approved by the Aoikai Clinic Ethics Committee (review number: 2022–01–07). All the procedures were performed in accordance with the ethical standards of the committee responsible for human experimentation and the Declaration of Helsinki. Informed consent was obtained from all the participants.

### Color-changeable chewing gum and its evaluation

When chewed, the color of the chewing gum (32 × 20 × 4 mm^3^; 3.0 g; Masticatory Performance Evaluating Gum XYLITOL, Lotte, Tokyo, Japan) changes from yellow-green to red. The gum base contains xylitol, citric acid, and red, yellow, and blue dyes that do not adhere to denture materials, allowing even complete denture wearers with reduced masticatory force to chew. Red dye is pH-sensitive and changes color in neutral or alkaline environments. Citric acid helps maintain the low internal pH of the yellow-green gum before chewing. The yellow and blue dyes soak into the saliva, and citric acid leaches out, changing the color of the gum to red as it is chewed. Changes in the color tone of the gum reflect the overall masticatory performance, including dental occlusion, saliva secretion, and tongue movement during chewing [7, 8, 10].

Color was assessed immediately after chewing the gum for 120 s. The chewed gum was spit out into a polyvinylidene chloride film. Subsequently, the gum in the film was compressed between two acrylic plates to a thickness of approximately 1.5 mm (to be between 1.0 and 2.0 mm) with a ruler as a guide and was measured using a colorimeter and color scale.

### Colorimeter measurement

**Fig. 1:**
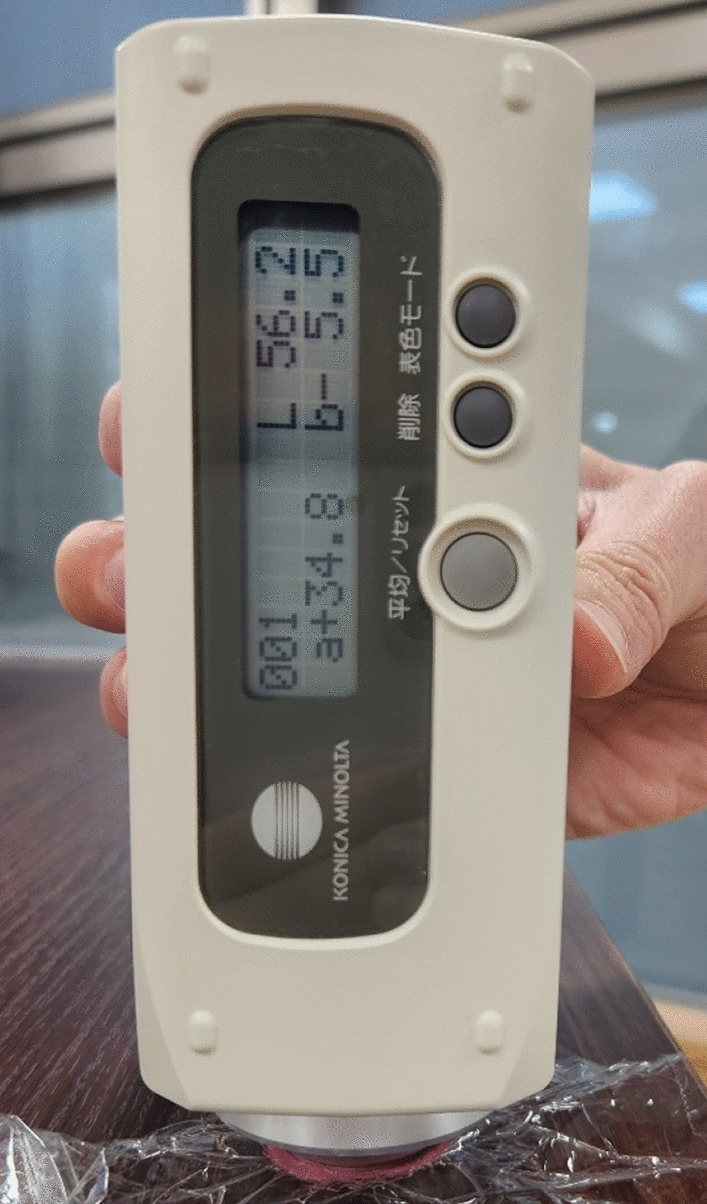
Colorimeter. The a* value indicates 34.8

Color changes were visualized using three-dimensional coordinates organized along the L*, a*, and b* axes and evaluated using the CIELAB color system established by the International Commission on Illumination (abbreviated as CIE from its French title). CIELAB color system is based on the Lab color space, which mathematically describes all perceivable colors in three dimensions: L* represents the lightness of the color, a* represents the degree of color between red and green, and b* represents the degree of color between yellow and blue. L*, a*, and b* are measured using a calorimeter (CR-13; Konica Minolta Sensing, Tokyo, Japan) (Fig. 1) and a positive value for a* indicates red, and a negative value for a* indicates green. The mean differences between two colors in the CIELAB color space (Δ*E*) were calculated before and after chewing using the following equation, in which the measured L*, a*, and b* values before chewing were 72.3, − 14.9, and 33.0, respectively: Δ*E* = $\sqrt{{(L* - 72.3)}^{2} + {(a* + 14.9)}^{2} + {(b* - 33.0)}^{2}}$. The Δ*E* value represents color changes before and after chewing and can be used to evaluate masticatory performance. The color-changeable chewing gum was evaluated by measuring only the a* value. For colorimetry, immediately after chewing for 120 s, the chewed gum was flattened with a polyvinylidene chloride film to a thickness of approximately 1.5 mm, and a colorimeter was placed at the center and approximately 5 mm from the center, top, bottom, left, and right, and measured at five points. An average of the five points was obtained for each a* value.

### Color scale measurement

**Fig. 2:**
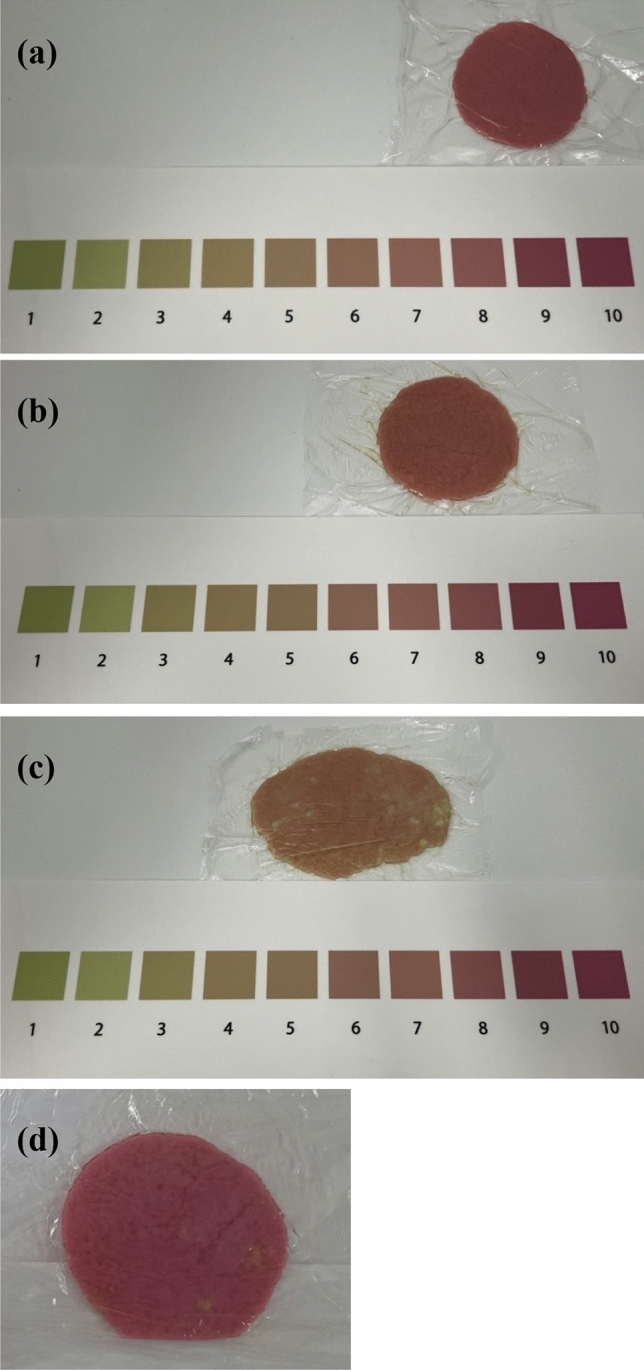
Gum color results. **a** Gum after chewing with uniform color (“A” with a* of 32.8 by the colorimeter, “a” on the color scale). **b** Gum after chewing with uniform color (“B” with a* of 25.1 on the colorimeter, “b” on the color scale). **c** Gum after chewing with green speckled in red (“C” with a* of 15.0 by the colorimeter, “c” on the color scale). **d** Gum after chewing with a few green dots mixed in with the red (“B” with a* of 26.0 by the colorimeter, “b” on the color scale)

The color change of the chewing gum was assessed immediately after chewing for 120 s using a color scale (Oral Care, Tokyo, Japan; Fig. 2a–c). The color scale comprised 10 colors (score 1–10). The scores increased as the color changed closer to red [7].

### Assessment

The cut-off value of a* measured using a colorimeter was classified into three groups: (A) masticable meat, > 28.7; (B) masticable soft side dish, 21.2–28.6; and (C) inadequate masticatory performance, < 21.1 based on a previous study [8]. The relationship between the color scale and color difference (Δ*E*) was proposed by Hama et al. [7]. For example, 7 on the color scale corresponds to a Δ*E* of 42. The equation a* = 0.75Δ*E*−14.59, which demonstrates the relationship between Δ*E* and a*, can be used to demonstrate the relationship between the color scale and a* [7]. Using this equation, 7 on the color scale corresponds to Δ*E* = 42, which is equivalent to a* of 16.91. Similarly, color scale 8 corresponds to a*22.16 and color scale 9 to a*27.41. Therefore, visual measurement of the color scale was performed using values that approximated the cut-off value of a* (10-point evaluation) and subsequently classified into three groups: (a) masticable meat: 9 ≤, (b) masticable soft side dish: 7 < and < 9, and (c) inadequate masticatory performance ≤ 7 (Table 1). If the color of the gum became speckled and it was difficult to judge the color, the masticatory performance was considered insufficient, with a score of 7 or less. For the color scale classification, one physiatrist and one dietitian visually judged the color change of the chewing gum simultaneously and individually and classified it into three groups: masticable meat, masticable soft side dish, and inadequate masticatory performance. Participants were categorized into three groups (A, B, and C) based on colorimeter measurements and the visual color scale (*a*, *b*, and *c*). We then verified whether the three-color scale groups were consistent with the colorimeter classification. Criterion-related validity was evaluated using the kappa coefficient of agreement between the results obtained using the two methods. Inter-rater reliability was examined using the kappa coefficient of agreement between the three groups as judged by the two raters on a color scale. The degree of agreement according to the value of the kappa coefficient is as follows: < 0.00 (poor), 0.00–0.20 (slight), 0.21–0.40 (fair), 0.41–0.60 (moderate), 0.61–0.80 (substantial), and 0.81–1.00 (almost perfect) [11].

**Table 1:** Relationship between colorimeter and color scale

	Colorimeter	Color scale number
Masticable meat	> 28.7	9 ≤
Masticable soft side dish	21.2–28.6	7 < and < 9
Inadequate masticatory ability	< 21.1	≤ 7

### Statistical analysis

The rationale for establishing the number of participants in the analysis as the sample size was as follows: the correlation coefficient between the value determined by the scale and the a* values measured by the reader was expected to be as high as 0.6–0.8. Assuming an alpha error rate (the probability of judging a correlation to be present when it is not) of 0.05 and a beta error rate (the probability of judging a correlation to be absent when it is present) of 0.20, the required number of participants was 10–19. However, since there were two examiners (*k* = 2), the 95% confidence interval width and correlation coefficient were estimated at 0.2 and 0.6, respectively, and the number of patients required was > 50 [12]. We determined that the required sample size for this study was 50. JMP version 16 (SAS Institute Inc., Cary, NC, USA) was used for all the analyses.

## Results

### Participants

From August to October 2022, 74 consecutive patients and daycare attendants of the Moriyama Rehabilitation Clinic were evaluated, 7 of whom were aged < 65 years; of the remaining 67 patients, one had poor general health due to an acute illness, and nine were unable to follow our instructions due to dementia. Of the remaining 57 patients, 50 were willing to participate in the study.

### Characteristics

Table 2 presents the demographic and clinical characteristics of the participants. The mean age ± standard deviation (SD) of the participants was 82.6 ± 7.8 years, and 24 participants (48%) were men. Half of the patients were hospitalized. Based on the metabolic disease components examined, 60% of the patients were hypertensive; 8% of the patients in this study had Parkinson’s disease. The following are the results of the simple tests for assessing swallowing function, with patient mean ± SD. The repetitive saliva swallowing test [13, 14], functional oral intake scale [15, 16], and Seirei dysphagia screening questionnaire [17] (answer of A was defined as dysphagia) provided scores of 2.2 ± 1.2, 6.2 ± 0.96, and 12, respectively. The Barthel Index [18], which assesses the patient’s ability to perform daily activities, such as eating and dressing, was 70.76 ± 26.26. The a* value of the color-changeable chewing gum after 120 s of chewing was 21.8 ± 11.6, which was nearly the same as the a* value (of 21.6 ± 11.6 [median: 27.8]) in the study that initially evaluated the cut-off value of a* [8]. Based on the occlusal support evaluation (Eichner classification) [19], 40%, 50%, and 10% of the patients were classified as A (all four occlusal support areas present), B (partial loss of occlusal support areas), and C (no support areas at all), respectively.

**Table 2:** Participant characteristics

Characteristic	Values are presented as the mean ± standard deviation (median) or *n* (%)
Age (years)	82.6 ± 7.8
Sex (male)	24 (48%)
Inpatient	25 (50%)
Hypertension	30 (60%)
Dyslipidemia	17 (34%)
Diabetes	8 (16%)
Stroke	16 (32%)
Parkinson’s disease, Parkinson’s syndrome	8 (16%)
Heart failure	5 (10%)
Eichner index (supporting zones with removable prostheses)	
A	20 (40%)
B	25 (50%)
C	5 (10%)
Repetitive saliva swallowing test	2.2 ± 1.2 (2)
Functional oral intake scale	6.2 ± 0.96 (6)
Dysphagia by Seirei dysphagia screening questionnaire^a^	12 (24%)
Barthel Index	70.76 ± 26.26 (80)
The a* values of the color-changeable chewing gum after 120 s of chewing	21.8 ± 11.6 (median: 27.4, first quartile: 19.55, third quartile: 30.15)

^a^“Answer of A” was defined as dysphagia by the Seirei dysphagia screening questionnaire

### Color scale

A typical example of visual evaluation using a color scale is shown in Fig. 2a–c. The colorimeter classified the patient’s gum in Fig. 2a as “A” with an a* of 32.8. The same patient was classified as “a” on the color scale. The patient’s gum in Fig. 2b was classified as “B” with a* of 25.1 on the colorimeter and as “b” on the color scale. The patient’s gum in Fig. 2c, which was red with green, was classified as “c” on the visual color scale. The a* value was 15.0 on the colorimeter, which was also classified as “C.” The following is an atypical example that is difficult to determine on the color scale: The gum in Fig. 2d was classified as “b” on the visual color scale, because it was entirely red, with a few scattered green tones. Because the a* value on the colorimeter was 26.0, it was classified as “B.”

Criterion-related validity was evaluated using the kappa coefficient of agreement between the three groups using the colorimeter (A, B, and C) and visual color scale (*a*, *b*, and *c*). One physiatrist and one dietitian made visual judgments using a color scale. The classification based on the color scale values was compared with the classification by a colorimeter (Table 3). The results demonstrated that the kappa coefficients of agreement for the classification were 0.908 and 0.909 for the physiatrist and dietitian, respectively (Table 4). Inter-rater reliability was examined using the kappa coefficient of agreement between the three groups as judged by the physiatrist and dietitian on a color scale (Table 3); the kappa coefficient was 0.938 (Table 4).

**Table 3:** Match/mismatch between the colorimeter and color scale and between the physiatrist’s and dietitian’s classifications

Color scale physiatrist’s classifications		Colorimeter		
		A	B	C
a		19	3	0
b		0	11	0
c		0	0	17

Color scale dietitian’s classifications		Colorimeter		
		A	B	C
a		19	2	0
b		0	11	0
c		0	1	17

Color scale dietitian’s classifications		Color scale physiatrist’s classifications		
		a	b	c
a		21	0	0
b		1	10	0
c		0	1	17

**Table 4:** Criterion-related validity and inter-rater reliability

	Kappa coefficient	Lower limits of the 95% confidence interval
Colorimeter and color scale physiatrist’s classifications	0.908	0.809
Colorimeter and color scale dietitian’s classifications	0.909	0.809
Color scale physiatrist and dietitian’s classifications	0.938	0.854

## Discussion

Visual inspection using a color scale can indicated masticable foods without using any specialized equipment. The color scale visual measurement method could be a helpful tool for assessing masticatory performance in a simple and location-independent manner.

This study found high agreement between the color scale and a colorimeter in assessing the masticatory performance. The inter-rater reliability was also high. Therefore, it was possible to determine masticable foods by visual assessment using an existing color scale. Herein, we provided interpretation guidelines in the form of color scale number thresholds: 9 ≤ indicates masticable meat, 7 < and < 9 indicates masticable soft side dish, and ≤ 7 indicates inadequate masticatory performance (Table 1). Since this method only requires visual evaluation of the color change, caregivers can easily determine masticable foods in medical institutions, older adult facilities, and at homes. Therefore, for older people who can eat only pureed food, this would reduce the amount of unnecessarily mechanically processed foods and provide them with safe and flavorful food.

The color scale visual measurement method can be easily performed at the bedside, which may contribute to building evidence for training as an outcome measure. In swallowing training, several studies and case reports on compensatory techniques, such as swallowing posture and voluntary swallowing techniques, and motor training, such as head raising, tongue resistance, and tongue retention training, have been published; however, the level of evidence for the usefulness of any of these techniques is not high [20–24]. Large-scale clinical studies are warranted to universalize individual training techniques and establish evaluation methods for their effectiveness. Finding effective methods with a high level of evidence for swallowing training is also important to determine the masticatory performance. The color scale visual measurement method may be helpful for masticatory performance assessment in a simple and location-independent manner.

The study has several limitations. First, the two raters were familiar with the evaluation, because it had already been clinically applied at our facility. Therefore, it is necessary to examine whether the same results would be obtained regardless of who performed the color scale evaluation. Second, it was difficult to evaluate the boundary area between A and B. In cases wherein the colorimeter and color scale results were inconsistent, the a* values in the A and B boundary regions were often between 27 and 28. The reason for this is that A in the colorimeter has an a* value of ≥ 28.7, while a value of ≥ 9 on the color scale has an a* value of 27.41; thus, a slight error exists, which makes the assessment difficult. Moreover, when the meat masticable group was defined as ≥ 9 (a* value of ≥ 27.41) on the color scale, the specificity was somewhat lower; therefore the positive predictive value was indirectly slightly low, and it is possible that certain individuals with poor meat mastication were classified as meat masticable [8]. In fact, all three of the mismatches evaluated by the physician and two of the three mismatches evaluated by the dietitian corresponded to this error. However, this study demonstrated that even if this error between 27.41 and 28.7 is set as an acceptable range, the agreement between the color scale and the colorimeter would be high; therefore, validity can be ensured. Although when evaluated near such a boundary, a comprehensive decision should be made using other assessments. Third, in some cases, the gum’s color was blurred after chewing, making classification of the masticatory performance challenging. A greenish mixture indicates that the mastication of a meal may be uneven. Thus, the mastication was inadequate, because there were unchewed areas (Fig. 2c). In contrast, if only a few scattered green areas were observed, it was unlikely that the green areas would decrease the a* value, since the color scale was judged by the color of the red areas (Fig. 2d). Finally, a previous study showed that for boiled rice, ginger-fried pork loin, boiled fish-paste, and rice cracker, the a* values of the color-changeable chewing gum after 120 s of chewing were significantly associated with suitable preparation of the food for swallowing. For sliced white bread, however, the a* values were not significantly associated with suitable preparation of the food for swallowing. The reason was that adhesion was too high (over 1000 J/m^2^), despite suitable hardness and cohesiveness [8]. Therefore, some ingredients require further evaluation.

Despite these limitations, this study’s results revealed high agreement between the color scale and colorimeter when assessing whether a certain food item was masticable and the inter-rater reliability was also high. Therefore, we believe that the color scale could be used in clinical application.

In conclusion, visual inspection using a color scale makes it possible to determine the masticable meal form without the use of special equipment, thus providing older people with safe and flavorful food. Since this method only requires visual evaluation of the color change, caregivers can easily determine masticable foods in medical institutions, older adult facilities, and at homes.

## Data Availability

The datasets used in this study are available from the corresponding author upon reasonable request.
